# Human Dendritic Cell DC-SIGN and TLR-2 Mediate Complementary Immune Regulatory Activities in Response to *Lactobacillus rhamnosus* JB-1

**DOI:** 10.1371/journal.pone.0120261

**Published:** 2015-03-27

**Authors:** Patrycja Konieczna, Elisa Schiavi, Mario Ziegler, David Groeger, Selena Healy, Ray Grant, Liam O’Mahony

**Affiliations:** 1 Swiss Institute of Allergy and Asthma Research, University of Zurich, Davos, Switzerland; 2 Alimentary Health Ltd., Cork, Ireland; 3 Alimentary Health Pharma Davos, Davos, Switzerland; Wayne State University, UNITED STATES

## Abstract

The microbiota is required for optimal host development and ongoing immune homeostasis. *Lactobacilli* are common inhabitants of the mammalian large intestine and immunoregulatory effects have been described for certain, but not all, strains. The mechanisms underpinning these protective effects are beginning to be elucidated. One such protective organism is *Lactobacillus rhamnosus* JB-1 (*Lb*. *rhamnosus* JB-1). *Lb*. *murinus* has no such anti-inflammatory protective effects and was used as a comparator organism. Human monocyte-derived dendritic cells (MDDCs) were co-incubated with bacteria and analysed over time for bacterial adhesion and intracellular processing, costimulatory molecule expression, cytokine secretion and induction of lymphocyte polarization. Neutralising antibodies were utilized to identify the responsible MDDC receptors. *Lb*. *rhamnosus* JB-1 adhered to MDDCs, but internalization and intracellular processing was significantly delayed, compared to *Lb*. *murinus* which was rapidly internalized and processed. *Lb*. *murinus* induced CD80 and CD86 expression, accompanied by high levels of cytokine secretion, while *Lb*. *rhamnosus* JB-1 was a poor inducer of costimulatory molecule expression and cytokine secretion. *Lb*. *rhamnosus* JB-1 primed MDDCs induced Foxp3 expression in autologous lymphocytes, while *Lb*. *murinus* primed MDDCs induced Foxp3, T-bet and Ror-γt expression. DC-SIGN was required for *Lb*. *rhamnosus* JB-1 adhesion and influenced IL-12 secretion, while TLR-2 influenced IL-10 and IL-12 secretion. Here we demonstrate that the delayed kinetics of bacterial processing by MDDCs correlates with MDDC activation and stimulation of lymphocytes. Thus, inhibition or delay of intracellular processing may be a novel strategy by which certain commensals may avoid the induction of proinflammatory responses.

## Introduction

It is well accepted that the gut microbiota plays a pivotal role in mucosal immune system development and ongoing homeostasis [1–3]. Moreover, a diverse gut microbiota contributes to appropriate digestion of dietary substances, protects against pathobiont expansion, provides nutrients for the host and ensures appropriate development of the mucosal barrier [4–6]. The mechanisms underpinning these protective responses are varied and include not only intact microbial cell interaction with host receptors, but also microbial metabolic activity, including short chain fatty acid production and bile degradation, which influences biological functions of the whole organism [7–9].

Crosstalk between the microbiota and the host is tightly regulated. Expression of pattern recognition receptors (PRRs) allows the immune system to discriminate between commensal and harmful microbes. Activation of diverse PRRs leads to differential cascades in signaling pathways and subsequently polarized secretion of cytokines and metabolites. Certain protective microbes interact with mucosal cells and contribute to the maintenance of mucosal tolerance [10–12]. In recent years, Lactobacilli strains have been repeatedly shown to have many beneficial effects within the gut [13]. Many diverse species and strains have been well characterized. *Lactobacillus rhamnosus* JB-1 (*Lb*. *rhamnosus* JB-1) has been well described as a tolerogenic commensal microbe, with multiple effects on mucosal cells including the induction of Foxp3+ T regulatory cells [14–16]. In addition, this microbe exerts potent effects on the gut-brain axis [17]. However, not all Lactobacillus strains exert the same effects, for example *Lactobacillus salivarius* AH102 does not induce T regulatory cells [18].

The molecular mechanisms underpinning the tolerogenic effects of Lactobacilli are diverse and are only beginning to be described in detail. Peptidoglycan-associated muropeptide produced by *Lactobacillus salivarius* Ls33 is thought to be responsible for its anti-inflammatory effect [19]. Lipoteichoic acid D-alanylation decreases anti-inflammatory responses associated with *Lactobacillus plantarum* and *Lactobacillus acidophilus* [20, 21]. Following removal of this modification, mutants were more efficient in their ability to protect against colitis in murine models. Moreover, secreted proteins such as p40 and p70 from *Lactobacillus rhamnosus* GG have been shown to enhance a functional epithelial barrier and improve cell survival [22–24]. Even small differences, such as glycosylation patterns, in cell-wall composition can lead to opposing effects on immune responsiveness [25, 26]. Indeed, the amount of a specific metabolite being secreted by a specific strain can have protective or pathological effects on the host immune system. For example, a *Lb*. *rhamnosus* strain, which secretes a low level of histamine, exerts protective immunological effects, while a *Lb*. *saerimneri* strain, which secretes high levels of histamine, induces an inflammatory response within the gut [27, 28]. However it is likely that many more, yet undiscovered, molecular mechanisms are involved and the data so far suggest that many of these mechanisms are bacterial strain and context dependent.

This diversity in molecular mechanisms is important, not least because mucosal recognition of bacterial strains via multiple PRRs on dendritic cells (DCs) will lead to divergent and polarized adaptive responses [29]. Typically, DCs will bind bacteria, internalize the microbe, lyse the bacterium within activated lysosomes and present the microbial antigens in the context of MHC. This response is accompanied by upregulation of costimulatory molecules and cytokine secretion in order to induce the appropriate tissue and adaptive immune responses. However, the combined examination of DC processing and cytokine secretion, in response to Lactobacilli strains, has not been previously described. In this report, we demonstrate an association between delayed DC intracellular processing of *Lb*. *rhamnosus* JB-1, which is associated with poor induction of costimulatory molecule expression and cytokine secretion, with preferential induction of Foxp3+ lymphocytes and no induction of T_H_1 or T_H_17 transcription factors. These responses are partially mediated via TLR-2 and DC-SIGN.

## Results

### MDDCs efficiently bind *Lb*. *rhamnosus* JB-1, but display delayed internalization and processing

**Fig 1:**
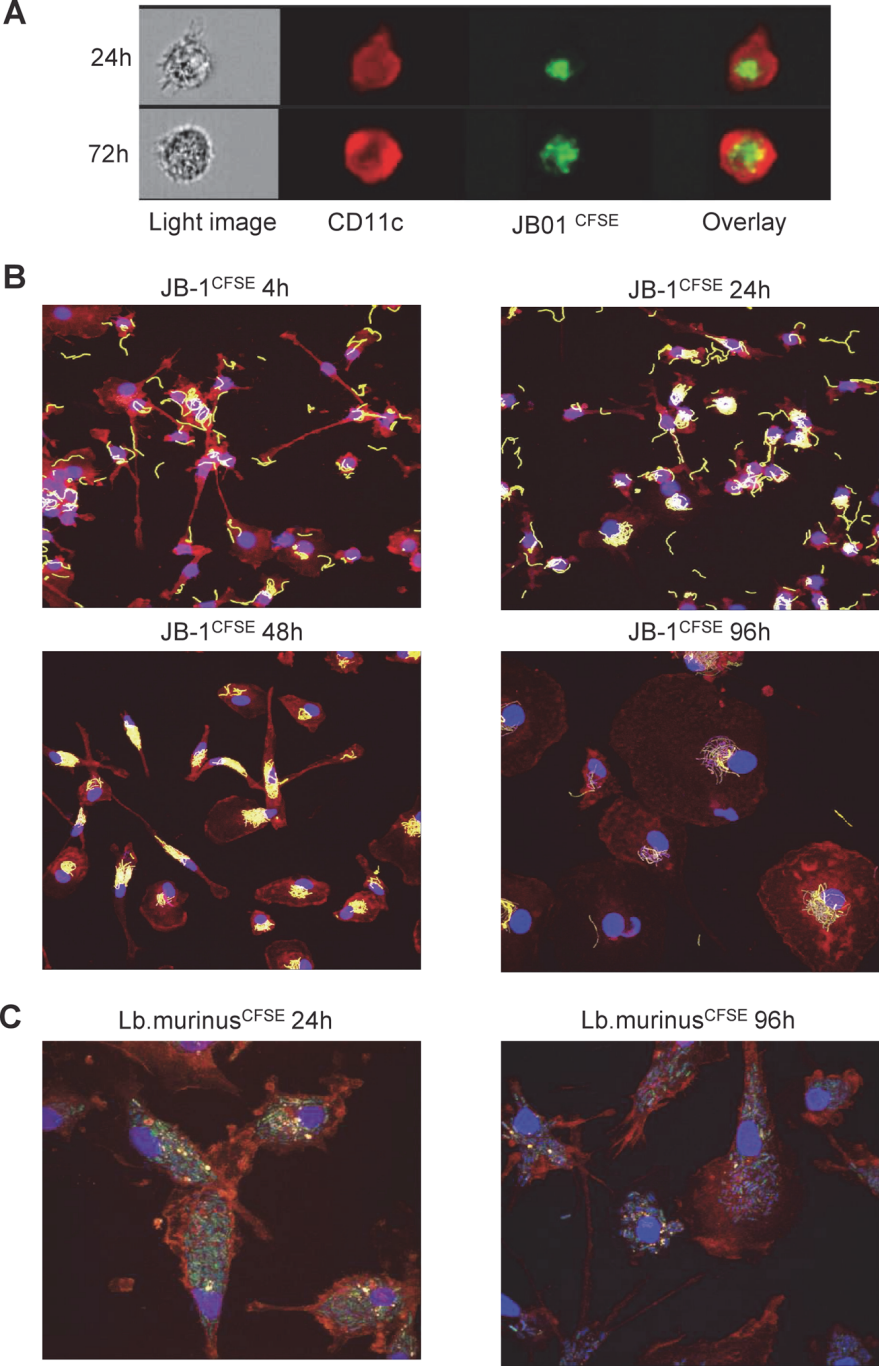
Differential MDDC binding and processing of two *Lactobacilli* strains. MDDCs bind *Lb*. *rhamnosus* JB-1 efficiently but intracellular processing is delayed, as visualized by multispectral flow cytometry imaging (A) and confocal microscopy (B). *Lb*. *murinus* is also efficiently bound by MDDCs and is rapidly processed (C). For both strains, bacterial cells are CFSE labeled (green), while CD11c^+^ cells are PE-Cy5 stained (red).

**Fig 2:**
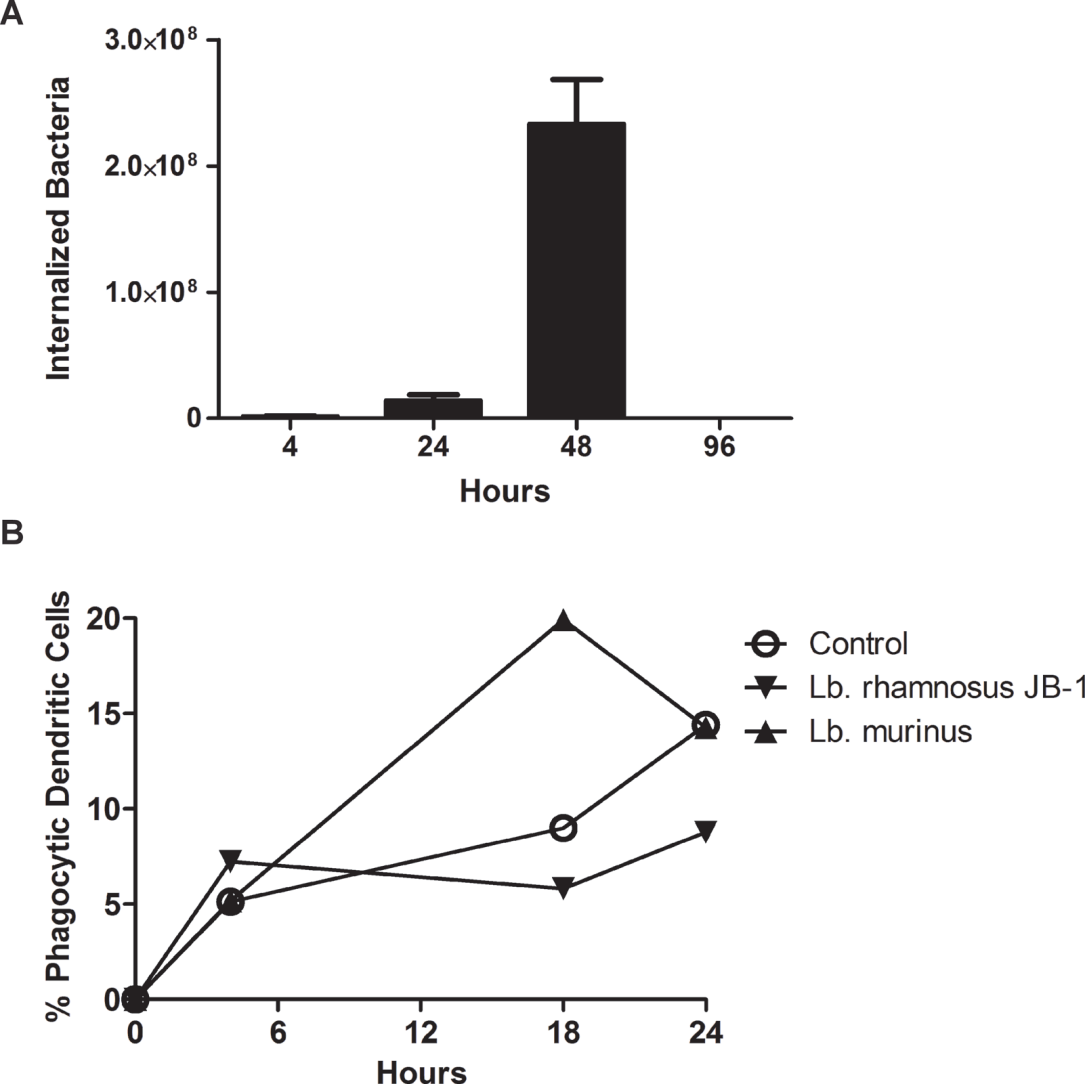
*Lb*. *rhamnosus* JB-1 internalisation by MDDCs. (A) Following antibiotic treatment, which kills bacteria outside MDDCs, the peak recovery of viable *Lb*. *rhamnosus* JB-1 was observed after 48 hours, suggesting that this was the time point when the bacteria were internalized by the MDDCs. (B) MDDC phagocytosis of another bacterial strain, *E*. *coli*, was reduced when the MDDCs were co-exposed to *Lb*. *rhamnosus* JB-1, but not *Lb*. *murinus*.

Human monocyte-derived dendritic cells (MDDCs) efficiently bind *Lb*. *rhamnosus* JB-1 as demonstrated by multispectral image stream analysis (Fig. 1A). In order to examine *Lb*. *rhamnosus* JB-1 internalisation and processing in more detail, we further examined MDDC responses to this microbe over an extended timeframe using confocal microscopy (Fig. 1B). Surprisingly, MDDCs did not internalize *Lb*. *rhamnosus* JB-1 for at least 48 hours following co-incubation, despite binding a large number of microbes on the cell surface. In addition, the binding pattern was unusual as distinct clustering of the bound microbe around the nucleus of the MDDCs was evident, even up to 96 hours following co-incubation. This delay in processing of *Lb*. *rhamnosus* JB-1 was not oberserved for an unrelated *Lb*. *murinus* strain (Fig. 1C), while the peri-nuclear binding was also not observed for *Lb*. *murinus*. Internalization of *Lb*. *rhamnosus* JB-1 was shown to peak at 48 hours as antibiotic treatment of the dendritic cells kills bacteria on the surface of the cells, while internalized bacteria are protected from the antibiotic allowing their growth and enumeration on agar plates (Fig. 2A). In addition, phagocytosis of fluorescently labeled *E*. *coli* was delayed in *Lb*. *rhamnosus* JB-1 treated dendritic cells, but not in *Lb*. *murinus* treated dendritic cells (Fig. 2B).

### Distinct MDDC responses are induced by two *Lactobacillus* strains

**Fig 3:**
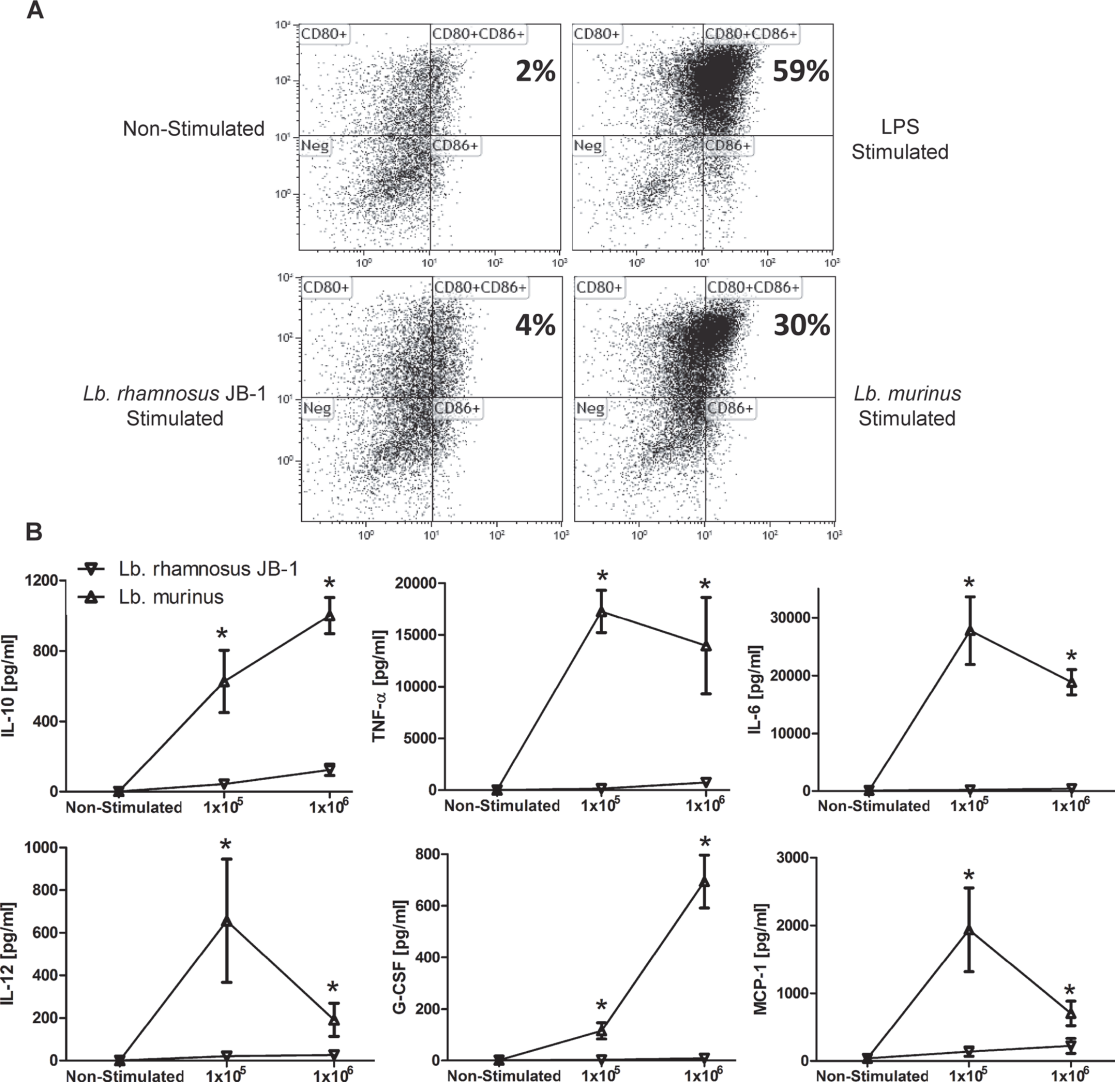
MDDC costimulatory molecule expression and cytokine secretion. (A) CD80 and CD86 were upregulated on MDDCs after exposure to both bacterial strains. LPS served as a positive control. Co-stimulatory molecule expression was significantly higher after stimulation with *Lb*. *murinus* compared with *Lb*. *rhamnosus* JB-1. (B) *Lb*. *murinus* stimulated MDDC cytokine secretion was significantly higher, for all cytokines tested, compared to that induced by *Lb*. *rhamnosus* JB-1 (n = 5 donors). *p<0.05

Following activation, MDDCs upregulate expression of cell surface co-stimulatory molecules and secrete cytokines. LPS stimulation resulted in robust stimulation of CD80/CD86 double positive MDDCs, followed by *Lb*. *murinus*, while *Lb*. *rhamnosus* JB-1 was a poor inducer of CD80/CD86 expression (Fig. 3A). *Lb*. *rhamnosus* JB-1-stimulated expression of CD80 and CD86 was significantly less than that induced by LPS or *Lb*. *murinus*.

MDDCs were co-incubated with 1x10^5^ or 1x10^6^ bacterial cells, and their supernatant cytokine levels were quantified after 24 hours. *Lb*. *murinus* induced high levels of TNF-α, IL-6, IL-10, IL-12p70, G-CSF and MCP-1. However, stimulation with *Lb*. *rhamnosus* JB-1 resulted in significantly less cytokine secretion, for all the cytokines measured (Fig. 3B). The relatively poor induction of co-stimulatory molecule expression and cytokine secretion by *Lb*. *rhamnosus* JB-1 is consistent with the imaging results, whereby the dendritic cell response to this bacterium seems to be minimal. SOCS3 has been previously described to suppress cytokine responses via negative regulation of MAP kinase activation. *Lb*. *rhamnosus* JB-1-stimulated MDDCs upregulated SOCS gene expression and p38 MAPK responses were significantly less than for LPS stimulated cells (S1 Fig.).

**Fig 4:**
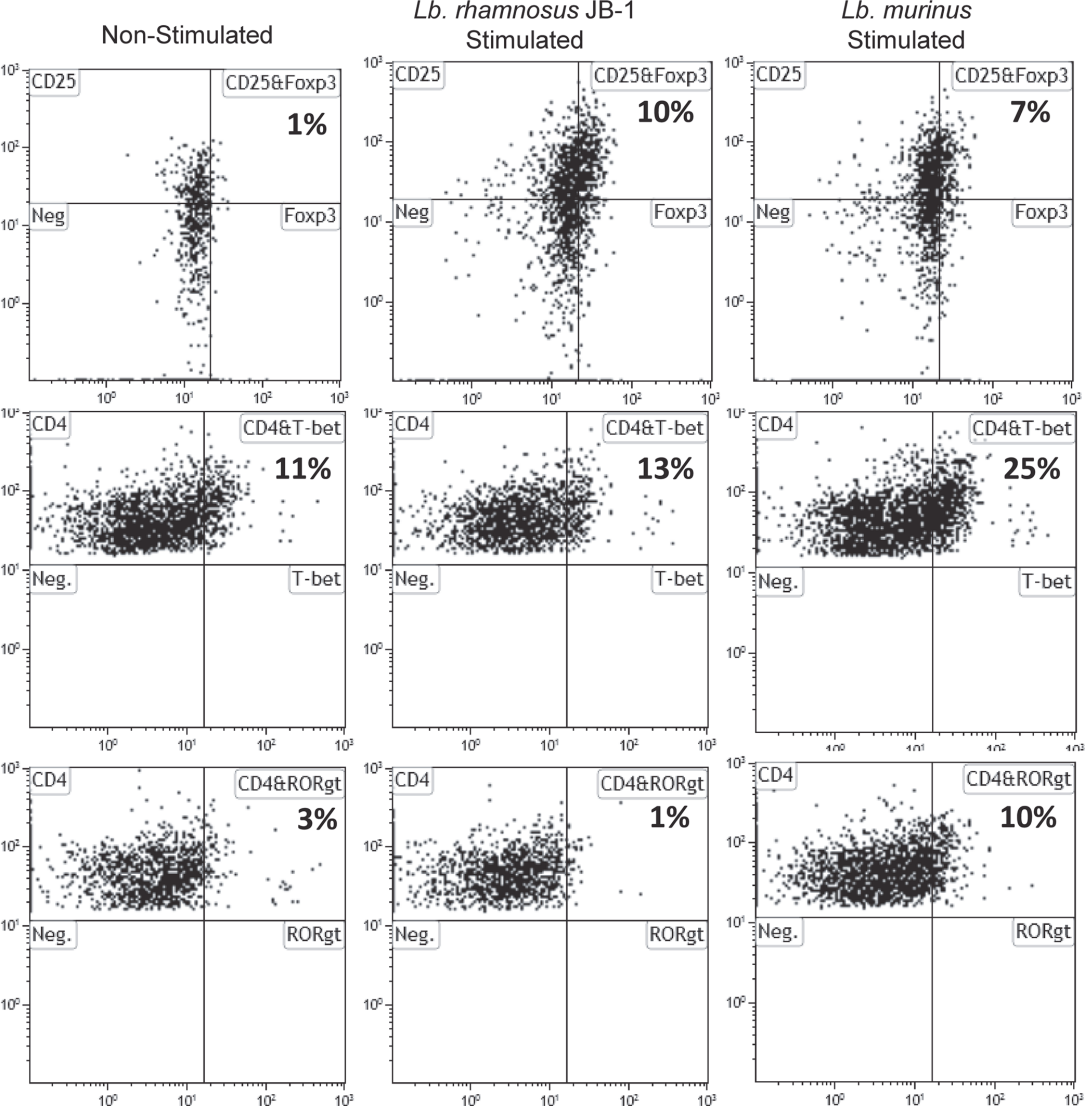
Transcription factor expression in lymphocytes co-incubated with bacterial primed MDDCs. Following 7 days co-incubation, MDDCs primed by both bacterial strains induced CD25^+^Foxp3^+^ T regulatory cells, while only MDDCs exposed to *Lb*. *murinus* increased T-bet and Ror-γt positive CD4^+^ T cells.

In order to further compare and contrast the dendritic cell response to these two lactobacilli strains, we co-incubated bacterial-stimulated dendritic cells with autologous lymphocytes for 7 days. Both *Lb*. *rhamnosus* JB-1 and *Lb*. *murinus*-stimulated MDDCs induced Foxp3 expression in autologous lymphocyte (Fig. 4). However, *Lb*. *murinus*-stimulated MDDCs also induced expression of T-bet (T_H_1) and ror-γt (T_H_17) transcription factors (Fig. 4). *Lb*. *rhamnosus* JB-1-stimulated MDDCs did not alter expression of the T_H_1 or T_H_17 transcription factors in autologous lymphocytes, when compared to transcription factor levels in control cultures (i.e. non-stimulated MDDCs and autologous lymphocytes co-incubation for 7 days).

### DC-SIGN, but not TLR-2, is required for *Lb*. *rhamnosus* JB-1 binding to MDDCs

**Fig 5:**
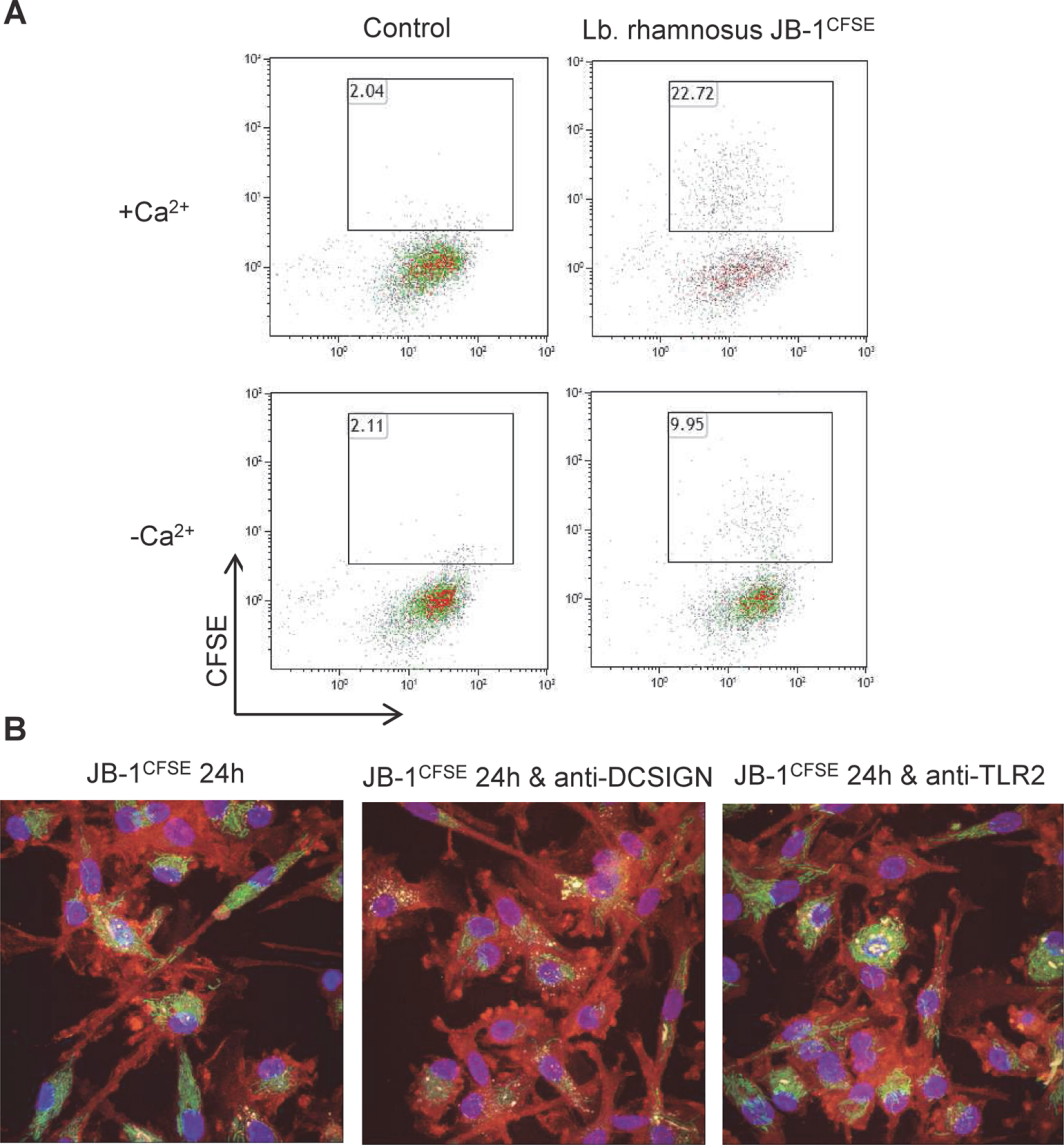
*Lb*. *rhamnosus* JB-1 is bound by MDDC DC-SIGN. (A) *Lb*. *rhamnosus* JB-1 binding to MDDCs is partially dependent on the presence of Ca^2+^ ions, suggesting the involvement of CLRs. The flow cytometry plots are representative of 3 independent experiments. (B) As visualized by confocal microscopy, blockade of DC-SIGN decreased *Lb*. *rhamnosus* JB-1 binding to MDDC, while neutralizing TLR-2 antibodies had no effect on *Lb*. *rhamnosus* JB-1 binding.

In order to further investigate the receptors that may be involved in MDDC recognition of *Lb*. *rhamnosus* JB-1, we specifically examined the role for DC-SIGN and TLR-2 as they have been previously shown by us and others to recognize commensal and pathogenic microbes. Firstly we assessed if c-type lectin receptors (CLRs) are required by comparing MDDC binding under calcium free conditions. MDDC capture of *Lb*. *rhamnosus* JB-1 was reduced by approximately 55% when calcium was absent from the medium suggesting that CLRs might be involved (Fig. 5A). DC-SIGN is a CLR previously described to recognize certain lactobacillus strains. When DC-SIGN was blocked using a neutralizing antibody, *Lb*. *rhamnosus* JB-1 adhesion to MDDCs was significantly reduced and the peri-nuclear clustering pattern was substantially reduced (Fig. 5B). However, neutralization of TLR-2 had no effect on *Lb*. *rhamnosus* JB-1 adhesion (Fig. 5B).

### *Lb*. *rhamnosus* JB-1 stimulation of MDDC cytokine secretion is DC-SIGN and TLR-2 dependent

**Fig 6:**
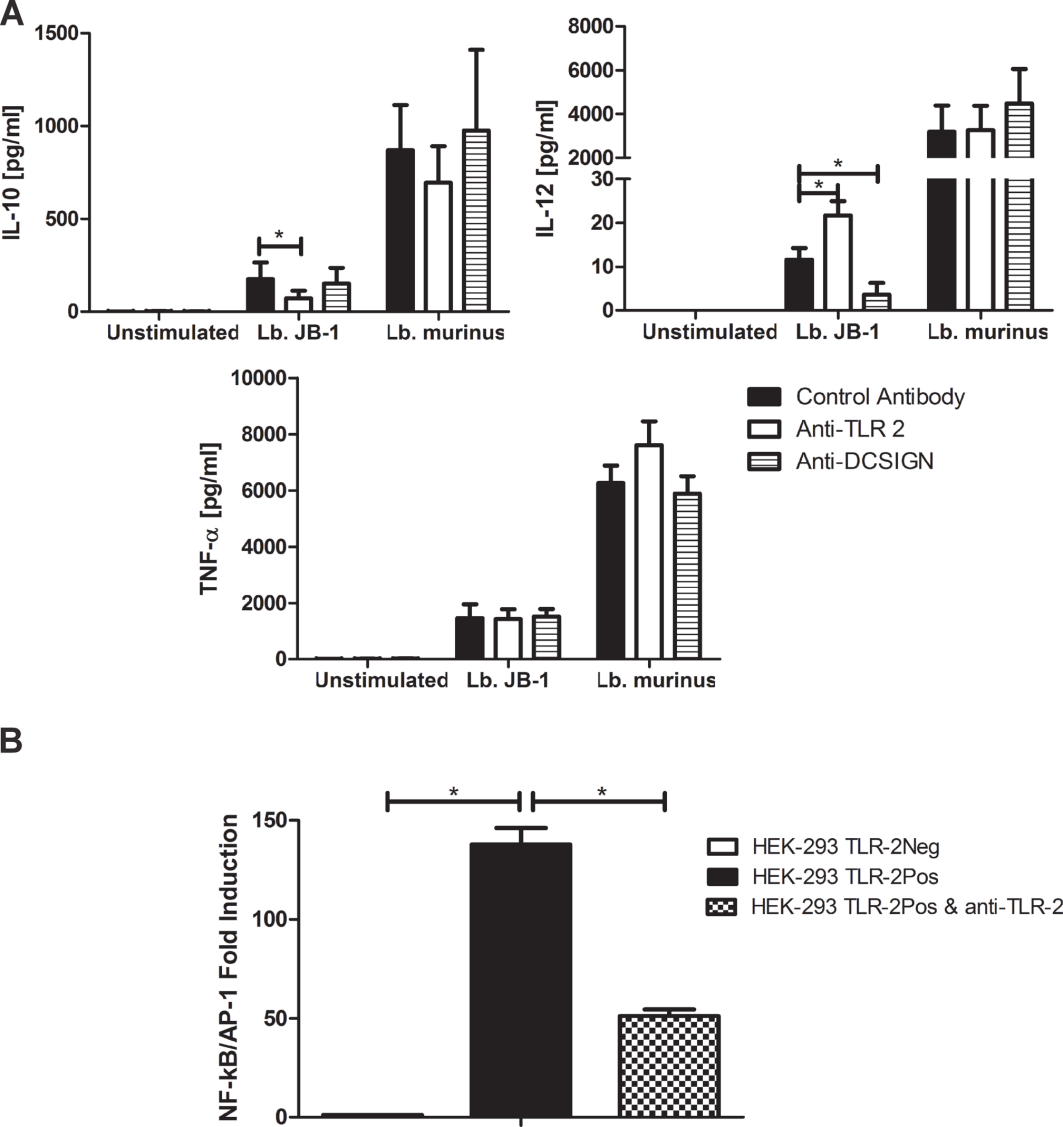
MDDC TLR-2 and DC-SIGN are required for appropriate cytokine responses to *Lb*. *rhamnosus* JB-1. *(A) Lb*. *rhamnosus* JB-1 or *Lb*. *murinus*-stimulated MDDCs were pre-incubated with blocking antibodies for TLR-2 (n = 8 donors), DC-SIGN (n = 8 donors) or antibody isotype controls for 30 minutes prior to stimulation and cytokine secretion was analyzed after 24 hours. Results are expressed as the mean pg/ml +/− standard error. (B) TLR-2 activation was confirmed using HEK-293 cells which express TLR-2. *p<0.05

MDDCs were pre-incubated with specific blocking antibodies for TLR-2 or DC-SIGN prior to bacteria stimulation and cytokine secretion was analyzed after 24 hours. Inhibition of TLR-2 signalling resulted in significantly decreased secretion of IL-10, associated with increased secretion of IL-12p70 in response to *Lb*. *rhamnosus* JB-1 (Fig. 6A). However, neutralization of TLR-2 activation did not alter the IL-10 or IL-12 response to *Lb*. *murinus* (Fig. 6A). Blockade of DC-SIGN had no effect on IL-10 or TNF-α secretion, while IL-12p70 secretion in response to *Lb*. *rhamnosus* JB-1 was significantly reduced. DC-SIGN neutralization had no effect on *Lb*. *murinus*-stimulated MDDC cytokine responses (Fig. 6A). We further confirmed that TLR-2 recognises *Lb*. *rhamnosus* JB-1 by utilizing HEK-293 cells, which express TLR-2. These cells responded to *Lb*. *rhamnosus* JB-1, as demonstrated by an increase in NF-κB/AP-1 activation, while neutralizing TLR-2 antibodies significantly reduced this response (Fig. 6B). In addition, TLR-2 deficient HEK-293 did not respond to *Lb*. *rhamnosus* JB-1 (Fig. 6B). MDDCs also express high levels of Dectin-1 and we performed blocking experiments for this receptor to investigate its role in cytokine secretion in response to *Lb*. *rhamnosus* JB-1. However, there was no significant difference in cytokine production following Dectin-1 neutralisation (data not shown).

## Discussion

In this report we demonstrate that the kinetics of DC processing and the magnitude of cellular activation induced by *Lb*. *rhamnosus* JB-1 are significantly different than that of another strain, *Lb*. *murinus*. At least two different PRRs are involved in this response, DC-SIGN and TLR-2. Even though *Lb*. *rhamnosus* JB-1 does not strongly stimulate DC responses, primed DCs are capable of inducing Foxp3 expression in lymphocytes. *Lb*. *murinus* primed DCs also induce Foxp3 expression but this is associated with T-bet and ror-γt expression.

These data suggest that one mechanism that may be exploited by *Lb*. *rhamnosus* JB-1 to prevent excessive inflammatory responses against this non-pathogenic microbe is to suppress DC activation. The data also suggest that this is an active process, rather than a passive process whereby the strain is simply not recognized by the DC. Blockade of TLR-2 results in less IL-10 being secreted, but more IL-12p70, a T_H_1 associated cytokine. In contrast, blockade of DC-SIGN results in reduced secretion of IL-12p70. This suggests that TLR-2 and DC-SIGN activation both influence different aspects of the DC response to *Lb*. *rhamnosus* JB-1.

TLR-2 is widely described as a PRR mediating host protective immune responses. In general, TLR-2^−/−^ DCs are not efficient in promoting Foxp3 Treg cell development and IL-10 secretion *in vitro* [30, 31]. Moreover, TLR-2-/- mice are more susceptible to DSS- induced colitis. TLR-2 activation by *Lb*. *rhamnosus* JB-1 is required for IL-10 secretion, while TLR-2 activation suppresses IL-12p70. The link between TLR-2 activation and suppression of IL-12 or CXCL-10 is unknown, however, SOCS3 gene expression is increased by *Lb*. *rhamnosus* JB-1-stimulated MDDCs. SOCS3 has been previously described to suppresses p38 MAPK and IL-12, IL-6, TNF-α and IL-23 secretion [32]. Thus SOCS3 induction by TLR-2 may be important for the low level of cytokine secretion induced by *Lb*. *rhamnosus* JB-1. Interestingly, not all lactobacilli strains can activate TLR-2, suggesting that strain-specific immunoregulatory effects may be partially mediated via TLR-2 activation [33].

DC-SIGN is a type II C-type lectin with a single carbohydrate recognition domain. DC-SIGN has been demonstrated to recognize a wide variety of pathogens and commensals including viruses such as HIV-1 and Ebola and bacteria such as *Mycobacterium tuberculosis*, *Lactobacillus casei* and *Bifidobacterium infantis* [34–36]. DC-SIGN recognizes mannose-capped lipoarabinomannan (ManLam) on Mycobacteria and activation of dendritic cells with ManLam leads to the generation of Foxp3+ Tregs involving a PGE2 dependent process [37]. DC-SIGN was previously shown to be important for lactobacillus primed MDDCs driven T regulatory cell development [38]. DC-SIGN and TLR-2 are required for IL-10 secretion and Foxp3 induction by another immunoregulatory microbe, *Bifidobacterium infantis* 35624 [35]. However, MDDCs process this Bifidobacterium strain very rapidly (less than 24 hours) suggesting that additional TLR-2/DC-SIGN independent *Lb*. *rhamnosus* JB-1-associated factors are responsible for the delayed intracellular processing and peri-nuclear clustering of this strain on human MDDCs.

A wide variety of *Lactobacilli* strains have been isolated from the human gastrointestinal tract and the majority have been shown to be commensal inhabitants of mammalian large intestine. However, certain strains have now clearly been demonstrated to exert additional beneficial effects on host immunoregulatory processes, such as that already described for *Lb*. *rhamnosus* JB-1. This microbe has shown protective effects in a range of murine models and we now describe certain potential cellular mechanisms underpinning this regulatory response. Manipulation of regulatory DC and T regulatory cell numbers or functions is an exciting therapeutic target in a wide range of inflammatory diseases [39, 40]. A clearer understanding of the mechanisms employed *in vivo* for the induction of oral tolerance by the microbiota will likely result in rational strategies to manipulate both regulatory and effector T cells, thereby influencing gastrointestinal disorders such as food allergy, eosinophilic esophagitis, irritable bowel syndrome and inflammatory bowel diseases.

## Materials and Methods

### Dendritic cell isolation and culture conditions

Monocytes and T cells were obtained from healthy volunteers. Human peripheral blood monocytes were isolated using CD14 positive isolation with the MACS system (Miltenyi Biotec, 130-050-201). Cells were cultured in cRPMI media (Life Technologies, 21875-091) with interleukin 4 1000U/ml (Novartis) and granulocyte macrophage colony stimulating factor (PeproTech, 300-03) 1000U/ml for 5–6 days in order to differentiate them into monocyte-derived dendritic cells (MDDCs). Human peripheral blood CD4 T cells were isolated using negative selection with the MACS system. Purified cells were cultured in AIM-V media (Life Technologies, 12055091). MDDCs were cultured in cRPMI and stimulated for 24 hours with different doses of *Lactobacillus rhamnosus* JB-1, *Lactobacillus murinus* (both gifts from the Alimentary Health culture collection, Cork, Ireland) or 0.5μg/ml LPS (Sigma- Aldrich). Cells were pre-incubated with the blocking monoclonal antibodies: anti-TLR2 antibody 4μg/ml (gift from C. Kirschning, Munich), anti-DC-SIGN 2μg/ml (AZDN1) (Beckman Coulter, A07406) or IgG2B control antibody, clone 20116 (R&D Systems Europe, MAB004) for 30 minutes. Cytokine secretion was examined by Bio-Plex multiplex suspension array (Bio-Rad Laboratories). The costimulatory molecules CD80 and CD86 expression on MDDCs were measured with anti-CD80 FITC and anti- CD86 PE (Beckman Coulter) by flow cytometry. MDDCs were incubated with 5x10^6^ bacteria for 4, 8, 12, 16, 20 or 24 hours and mRNA was isolated by use of RNeasy Mini kit (Qiagen, Venlo, Netherlands). Reverse transcription was performed with Fermentas reagents, including hexamer primers (St. Leon-Rot, Germany). Real time PCR was performed with iTaq SYBR Green Supermix with ROX (Bio-Rad Laboratories, Hercules, USA) and primers: EF1a Fw: CTGAACCATCCAGGCCAAAT, EF1a Rv: GCCGTGTGGCAATCCAAT; SOCS3 Fw: TGTTTACAATCTGCCTCAATCACTCT, SOCS3 Rv: TCAAGCATCTCCTAATAGCCTCAA, synthesized by Microsynth (Balgach, Switzerland).

### Phagocytosis assay

MDDCs were plated at a density of 2x10^6^ per well in a 6-well plate. They were incubated alone or with *Lactobacillus rhamnosus* JB-1 or *Lactobacillus murinus* at 37°C, 5% CO_2_ for 4 hours to 24 hours. Bacteria were added at a 1:1 ratio with MDDCs. After the incubation period, MDDCs were washed with PBS, harvested and incubation with pHrodo Green E. coli BioParticles Conjugate for Phagocytosis (Molecular Probes) was performed. Flow cytometry was performed using Galios flow cytometer (Beckman Coulter). Kaluza software (Beckman Coulter) was used for the analysis of pHrodo Green E. coli BioParticles Conjugate positive cells.

### Internalization assay

MDDCs were incubated alone or with *Lactobacillus rhamnosus* JB-1 at 37°C, 5% CO_2_ for 4 hours to 96 hours. Bacteria were added at a 10:1 ratio with MDDCs. After the incubation period, gentamicin (100μg/ml) was added to kill extracellular bacteria. After 2 hours, the supernatant was removed, the cells were washed with PBS and 1% Triton X-100 was used to lyse dendritic cells and allow the release of internalized bacteria. Plating of serial dilutions on MRS agar (Merck, VWR International, Dietikon, Switzerland) followed by conventional plate counting was performed.

### MDDC-T cell co-cultures

MDDCs were stimulated for 4 hours with *Lactobacillus rhamnosus* JB-1 or *Lactobacillus murinus* and co-cultured with T cells from autologous donors at a ratio of 1:20 in AIM-V media. After 5 days cells were stimulated with CDMix: anti-human CD28 (generated in house), anti-human CD3 (Orthoclone OKT3, Janssen-Cilag) and anti-human CD2 (CLB-T11.2/1 and CLB-T11/1, Sanquin, M1652, M1651). Two days later cells were removed from culture and stained for CD4, CD25, Foxp3, T-bet and Ror-γt expression using flow cytometry (eBioscience, San Diego).

### Bacteria labeling and visualization

Bacteria were stained with carboxyfluorescein diacetate, carboxyfluorescein succinimidyl ester (CFSE) (Life technologies, C1157), while MDDCs were stained with anti-human CD11c PE-Cy5, clone B-ly6 (BD Pharmingen). MDDCs were incubated with CFSE-labeled bacteria for 4 hours in HBSS with or without calcium and magnesium. Binding was analyzed flow cytometry. Moreover, bacteria binding in cRPMI was visualized after 24 and 72 hours by use of multispectral imaging flow cytometer Image Stream X (Amnis Corporation) and images were analyzed with IDEAS software (Amnis Corporation). MDDCs were plated on round cover slips and incubated with bacteria for 4, 24, 48 or 96 hours with or without blocking monoclonal antibodies. Cells were additionally stained with DAPI in ProLong Gold antifade reagent (Life Technologies, P36935). Slides were analyzed by confocal microscopy.

### HEK-293 cells

HEK-Blue hTLR2 cells and HEK-Blue Null1 cells (Invivogen) were pre-incubated with anti-TLR2 antibody for 30 minutes and were stimulated with 1x10^5^ bacteria for 24 hours. Both cell lines express NF-κB-inducible secreted embryonic alkaline phosphatase (SEAP). NF-κB induction was measured indirectly by quantifying the enzymatic reaction with Quanti blue reagent (Invivogen, rep-qb1) and spectrophotometry.

### Statistical analysis

Unpaired student t-tests were used to compare bacterial-stimulated MDDC cytokine secretion levels. Wilcoxon matched-pairs test was used to evaluate the effect of blocking antibodies on activated dendritic cells. All data analysis was performed using GraphPad Prism software.

## Supporting Information

S1 Fig *Lb*. *rhamnosus* JB-1-stimulated MDDCs upregulated SOCS gene expression and p38 MAPK responses were significantly less than for LPS stimulated cells. (TIF)
