# Supplementary figures and images for: Human Dendritic Cell DC-SIGN and TLR-2 Mediate Complementary Immune Regulatory Activities in Response to Lactobacillus rhamnosus JB-1
Source: PLoS One. 2015 Mar 27;10(3):e0120261. doi: 10.1371/journal.pone.0120261 (PMC4376398; doi:10.1371/journal.pone.0120261)

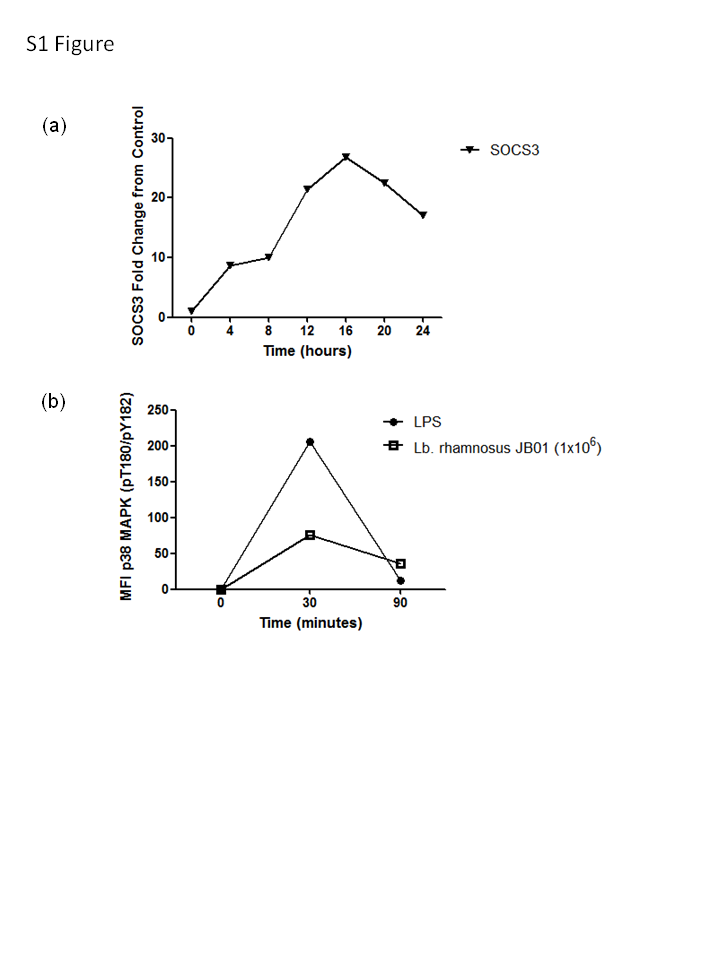

Supplement: S1 Fig — (TIF) [file pone.0120261.s001.tif]
